# Treatment patterns of patients with HR+/HER2- metastatic breast cancer receiving CDK4/6 inhibitor-based regimens: a cohort study in the French nationwide healthcare database

**DOI:** 10.1007/s10549-023-07201-w

**Published:** 2024-01-11

**Authors:** Stephanie H. Read, Nadia Quignot, Raissa Kapso-Kapnang, Erin Comerford, Ying Zheng, Corona Gainford, Medha Sasane, Anne-Lise Vataire, Laure Delzongle, Francois-Clement Bidard

**Affiliations:** 1 Certara UK Limited, London, UK; 2 Certara France, Paris, France; 3 Sanofi, Cambridge, MA USA; 4 Sanofi, Paris, France; 5 Department of Medical Oncology, Institut Curie, Saint-Cloud, France; 6 Université Versailles Saint-Quentin, Université Paris-Saclay, Saint-Cloud, France

**Keywords:** Hormone receptor negative, Metastatic breast cancer, Endocrine therapy, Cyclin-dependent kinase 4/6 inhibitor, Real-world evidence, SNDS

## Abstract

**Purpose:**

To assess real-world treatment patterns in patients diagnosed with hormone receptor positive (HR+), human epidermal growth factor receptor 2 negative (HER2-) metastatic breast cancer (mBC) who received cyclin-dependent kinase 4/6 (CDK4/6) inhibitors in combination with an aromatase inhibitor (AI) or fulvestrant at first line.

**Methods:**

Patient characteristics, treatment history, and outcomes data were extracted from the French ‘Système National des Données de Santé’ (SNDS) database for patients diagnosed with HR+/HER2- mBC between January 2014 and June 2019 and who received combination therapy with a CDK4/6 inhibitor and endocrine therapy. Kaplan-Meier methodology was used to assess time to next treatment (TTNT) and time to treatment discontinuation (TTTD).

**Results:**

The cohort comprised 6061 patients including 4032 patients who received CDK4/6 inhibitors + AIs and 2029 patients who received CDK4/6 inhibitors + fulvestrant. Median follow-up was 13.5 months (IQR 9.5–18.1). The median TTTD of first line treatment with CDK4/6 inhibitors + AIs and CDK4/6 inhibitors + fulvestrant was 17.3 months (95% CI 16.8–17.9) and 9.7 months (95% CI 9.0–10.2), respectively. Chemotherapy was the most common second line therapy. Median TTTD of subsequent treatment lines was progressively shorter following first line treatment with CDK4/6 inhibitors + AIs (2nd line: 4.6 months (95% CI 4.4–4.9) and with CDK4/6 inhibitors + fulvestrant (2nd line: 4.7 months (95% CI 4.3–5.1). TTNT was longer than TTTD across lines of therapy.

**Conclusion:**

This real-world analysis confirms the effectiveness of CDK4/6 inhibitor-based regimens in French patients and highlights the frequent use of chemotherapy as second line therapy.

**Supplementary Information:**

The online version contains supplementary material available at 10.1007/s10549-023-07201-w.

## Introduction

Globally, breast cancer is the most commonly diagnosed cancer and the leading cause of cancer-related death among women [1, 2]. Metastatic breast cancer (mBC) represents approximately 12% of all breast cancers in France, including those with metastatic disease at diagnosis and those that relapse [3, 4]. Hormone receptor positive/human epidermal growth factor receptor 2 negative (HR+/HER2-) breast cancer is the most common molecular subtype, accounting for 66% of all mBC cases [5].

Beyond chemotherapy, treatment options for HR+/HER2- mBC have evolved over the past several decades and include aromatase inhibitors (AIs), selective estrogen receptor modulators (SERMs) and selective estrogen receptor degraders (SERDs) as monotherapies or in combination with cyclin-dependent kinase 4/6 (CDK4/6) inhibitors, phosphatidylinositol-3-kinase (Pi3K) inhibitors or mammalian target of rapamycin (mTOR) kinase inhibitors [6]. International clinical guidelines recommend CDK4/6 inhibitors in combination with endocrine therapy (ET: AI or fulvestrant) as standard of care first line treatment for patients with HR + /HER2- mBC [7, 8].

The introduction of CDK4/6 inhibitors after 2015 changed the treatment landscape and improved outcomes among patients with HR+/HER2- mBC. Clinical trials have shown that CDK4/6 inhibitors in combination with ET are superior to ET alone with respect to progression-free survival (PFS) and overall survival [9–15]. For example, treatment with ET combined with ribociclib was demonstrated to improve PFS compared to ET alone among patients with HR+/HER2- mBC (20.5 months (95% confidence interval (CI): 18.5 to 23.5 months) vs.12.8 months (95% CI 10.9–16.3 months). Once patients progress on CDK4/6 inhibitors, treatment options are more limited and efficacy diminishes [9].

While the use of CDK4/6 inhibitors in HR+/HER2- mBC patients has increased over time since their introduction in France, there remains limited real-world data describing treatment patterns and outcomes in the post-CDK4/6 inhibitor era [16]. This study evaluated patient characteristics, treatment patterns, and outcomes among patients with HR+/HER2- mBC and who received CDK4/6 inhibitors combined with fulvestrant or AIs in first line in France.

## Methods

### Study design and data source

Data were obtained for this retrospective cohort study from the French ‘Système National des Données de Santé’ (SNDS) database. This nationwide data system collates data for over 65 million insurees, including data for inpatient and outpatient encounters by combining data from three databases using a unique social security number: ‘Système National d’Information Inter-Régime de l’Assurance Maladie’ (SNIIRAM), ‘Programme de Médicalisation des Systèmes d’Information’ (PMSI) and Epidemiological Center for the Medical Causes of Death database (CépiDc). SNIIRAM is the national health insurance claims database which collates patient demographic data and data related to medical care administered, including medications administered, procedures and laboratory tests undertaken. PMSI is a hospital discharge database which captures inpatient data including diagnoses based upon International Classification of Diseases-10th revision (ICD-10) codes. CépiDc is a death registry. Variables extracted from each database are presented in Supplementary Table S1.

This study was approved by the French ‘Commission Nationale Informatique et Libertés’ (CNIL) governing the data access and the data privacy laws.

### Study population

Patients were eligible for inclusion if they were adults (≥ 18 years) newly diagnosed with HR+/HER2- mBC between January 1st, 2014 and June 30th, 2019 and received fulvestrant or an AI (letrozole, anastrozole, exemestane) with a CDK4/6 inhibitor (palbociclib, ribociclib, abemaciclib) as first line mBC treatment. HR+/HER2- mBC diagnoses in breast cancer patients (ICD-10 code: C50) were ascertained using the following algorithm:

- Patients who had at least one occurrence of an Anatomical Therapeutic Chemical (ATC) code for targeted therapy: CDK4/6 inhibitors or fulvestrant or everolimus
  OR
- At least one occurrence of a diagnosis for ‘secondary malignant neoplasm’ (ICD-10 codes: C77*, C78*, C79*, excluding C77.3 and C79.2) followed by at least one occurrence of an ATC code for tamoxifen or an AI
  AND
- Patients without an ATC code for treatment targeting HER2+ (trastuzumab, ado-trastuzumab emtasine, afatinib, pertuzumab, lapatinib, neratinib, ado-trastuzumab emtansine (T-DM1))

Patients were excluded if they were diagnosed with other primary malignancies (excluding breast cancer) prior to mBC diagnosis, participated in a clinical trial, or if they had missing or invalid data.

Index date was set to the first occurrence of either a prescription record of a mBC drug (fulvestrant, everolimus or CDK4/6 inhibitors) or an ICD-10 diagnostic code for metastatic disease (C77*, C78*, C79*, excluding C77.3 and C79.2). Data for three years prior to index date was evaluated to identify first evidence of metastatic disease and to describe patient comorbidities and treatment history. Patients were followed from the index date until December 31st, 2019 (data cut-off date), date of last available record in SNDS, or date of death, whichever occurred earliest.

### Regimens and line of therapy

An algorithm was used to define line of therapy (LOT). A full description of the algorithm is provided in Supplementary Table S2. Briefly, a first-line regimen was defined as the first anticancer treatment(s) a patient receives after mBC diagnosis, including all eligible drugs observed within 30 days of the first eligible treatment. The first-line regimen would begin at the start date of the first medication in the combination and would end at the earliest of treatment augmentation, treatment switch, treatment discontinuation, death, last date of continuous enrolment or date of end of data availability. AIs and CDK4/6 inhibitors were exchangeable (i.e., switching between AIs and CDK4/6 inhibitors did not trigger a new line). Subsequent LOTs were defined similarly.

### Clinical characteristics

Visceral disease was defined as the presence of ICD-10 diagnostic codes for liver, lung/pleura, peritoneum, adrenal gland, ovary, or brain metastases [17]. Charlson comorbidity index was estimated by identifying ICD-10 diagnostic codes for relevant comorbidities (Supplementary Table S3) [18]. Patients defined as endocrine sensitive included those who had not received an AI or tamoxifen in the 12 months prior to first evidence of mBC while endocrine resistant patients were defined as patients who had received an AI or tamoxifen in this timeframe. Patients aged 50 years or older at index date were defined as post-menopausal and patients aged younger than 50 years were defined as pre-menopausal.

### Treatment patterns

Time to treatment discontinuation (TTTD) was assessed from initiation of treatment line until treatment discontinuation or death, whichever occurred earliest. Patients still on treatment at the end of the study period were censored on December 31st, 2019. Time to next treatment (TTNT) was assessed from initiation of treatment line to subsequent line of therapy initiation. Patients without subsequent LOT were censored at date of death or end of follow-up, whichever occurred earliest.

### Statistical analyses

Patient characteristics were described using descriptive statistics (n, mean, standard deviation (SD), median, interquartile ranges (IQR)) overall and according to whether the patient received fulvestrant or an AI in combination with the CDK4/6 inhibitor agent. Kaplan–Meier methodology was used to assess TTTD and TTNT. All statistical analyses were conducted using SAS (version 9.4).

## Results

### Baseline characteristics

There were 6061 patients with HR+/HER2- mBC who received combination therapy with either CDK4/6 inhibitors + AIs or CDK4/6 inhibitors + fulvestrant as first line mBC treatment between January 1st 2014 and June 30th 2019 (Supplementary Fig. S1). Among them, 4032 (67%) patients received CDK4/6 inhibitors + AIs and 2029 (33%) patients received CDK4/6 inhibitors + fulvestrant (Table 1). Overall, the median age at first evidence of mBC diagnosis was 66.5 (IQR 56.4–74.3) years, 15.1% of patients had evidence of visceral disease and less than 1% of the patients were male. Among female patients, 17.3% and 8.8% of patients who received CDK4/6 inhibitors + AIs and CDK4/6 inhibitors + fulvestrant were pre-menopausal (age < 50 years), respectively. Median follow-up time from first evidence of mBC was 13.5 months (IQR 9.5–18.1) and was similar among patients who received an AI or fulvestrant in combination with a CDK4/6 inhibitor (13.4 months (IQR 9.5–17.9) vs. 13.6 months (IQR 9.3–18.4)), respectively. Overall, 756 patients (12.5%) died during the follow-up period, including 9.4% of patients who received CDK4/6 inhibitors + AIs and 18.5% of patients who received CDK4/6 inhibitors + fulvestrant.

**Table 1:** Baseline demographic and clinical characteristics

	Overall	Patients who received CDK4/6 inhibitors + AI	Patients who received CDK4/6 inhibitors + fulvestrant
	(*N* = 6061)	(*N* = 4032)	(*N* = 2029)
Calendar year at first evidence of mBC diagnosis, *N* (%)			
2014	< 10	< 10	< 10
2015	< 10	< 10	< 10
2016	24 (0.4)	7 (0.2)	17 (0.8)
2017	342 (5.6)	134 (3.3)	208 (10.3)
2018	3531 (58.3)	2389 (59.3)	1142 (56.3)
2019 (January 1st–June 30th)	2151 (35.5)	1493 (37.0)	658 (32.4)
Age at time of first evidence of mBC			
Median (IQR)	66.5 (56.4–74.3)	65.4 (54.2–73.6)	68.0 (59.4–75.6)
Min: Max	24.7: 101.9	24.7: 96.2	25.6: 101.9
Female, *N* (%)	6019 (99.3)	4002 (99.3)	2017 (99.4)
Evidence of visceral metastases prior to and including index date, *N* (%)^a^	915 (15.1)	720 (17.9)	195 (9.6)
Menopausal/post-menopausal at first evidence of mBC (based upon age ≥ 50), *N* (%)	5149 (85.6)	3310 (82.7)	1839 (91.2)
CharIson Comorbidity Category^b^, *N* (%)			
0	2947 (48.6)	2136 (52.9)	811 (39.9)
1–2	1784 (29.4)	1125 (27.9)	659 (32.5)
3–4	380 (6.3)	228 (5.7)	152 (7.5)
≥ 5	950 (15.7)	543 (13.5)	407 (20.1)

^a^Liver, lung/pleura, peritoneum, adrenal gland, ovary, brain metastases

^b^Excluding cancer diagnosis

### Treatment history

There were more endocrine resistant than endocrine sensitive patients in the overall study population (52.2 vs. 47.8%) **(**Table 2**)**. Endocrine sensitive patients were more likely to receive CDK4/6 inhibitors + AIs in first line than CDK4/6 inhibitors + fulvestrant (61.8 vs. 20.2%). In the one-year prior to index date, 43.9% of all patients received an AI, 11.3% of patients received tamoxifen and 9.1% received chemotherapy (patients could have received more than one type of therapy). A smaller proportion of patients who received CDK4/6 inhibitors + AIs received an AI in the one-year prior to index compared to patients who received CDK4/6 inhibitors + fulvestrant (29.6% vs.72.3%). The proportion of patients who received tamoxifen in the one-year prior to index date was similar across CDK4/6 inhibitor-based regimens (CDK4/6 inhibitors + AI: 11.5%, CDK4/6 inhibitors + fulvestrant: 11.1%).

**Table 2:** Treatment history one year prior to index date

Cancer treatments one year prior to index date	Overall	Patients who received CDK4/6 inhibitors + AI	Patients who received CDK4/6 inhibitors + fulvestrant
	(*N* = 6061)	(*N* = 4032)	(*N* = 2029)
Any cancer medication, *N* (%)	3370 (55.6)	1668 (41.4)	1702 (83.9)
AI, *N* (%)	2659 (43.9)	1192 (29.6)	1467 (72.3)
Tamoxifen, *N* (%)	687 (11.3)	462 (11.5)	225 (11.1)
Chemotherapy *N* (%)	554 (9.1)	274 (6.7)	280 (13.8)
Radiotherapy	148 (2.4)	81 (2.0)	67 (3.3)
Surgery, *N* (%)	407 (6.7)	331 (8.2)	76 (3.8)
No evidence of AI or tamoxifen (proxy for endocrine sensitivity)	2899 (47.8)	2490 (61.8)	409 (20.2)

The proportion of patients who received chemotherapy pre-index was lower among patients who received CDK4/6 inhibitors + AIs than among patients who received CDK4/6 inhibitors + fulvestrant (6.7 vs. 13.8%).

### Treatment patterns and outcomes during follow-up

The mean number of LOTs observed during follow-up was 1.4 (SD: 0.8) and 1.8 (SD: 1.1) in patients who received CDK4/6 inhibitors + AIs and in patients who received CDK4/6 inhibitors + fulvestrant, respectively.

**Fig. 1:**
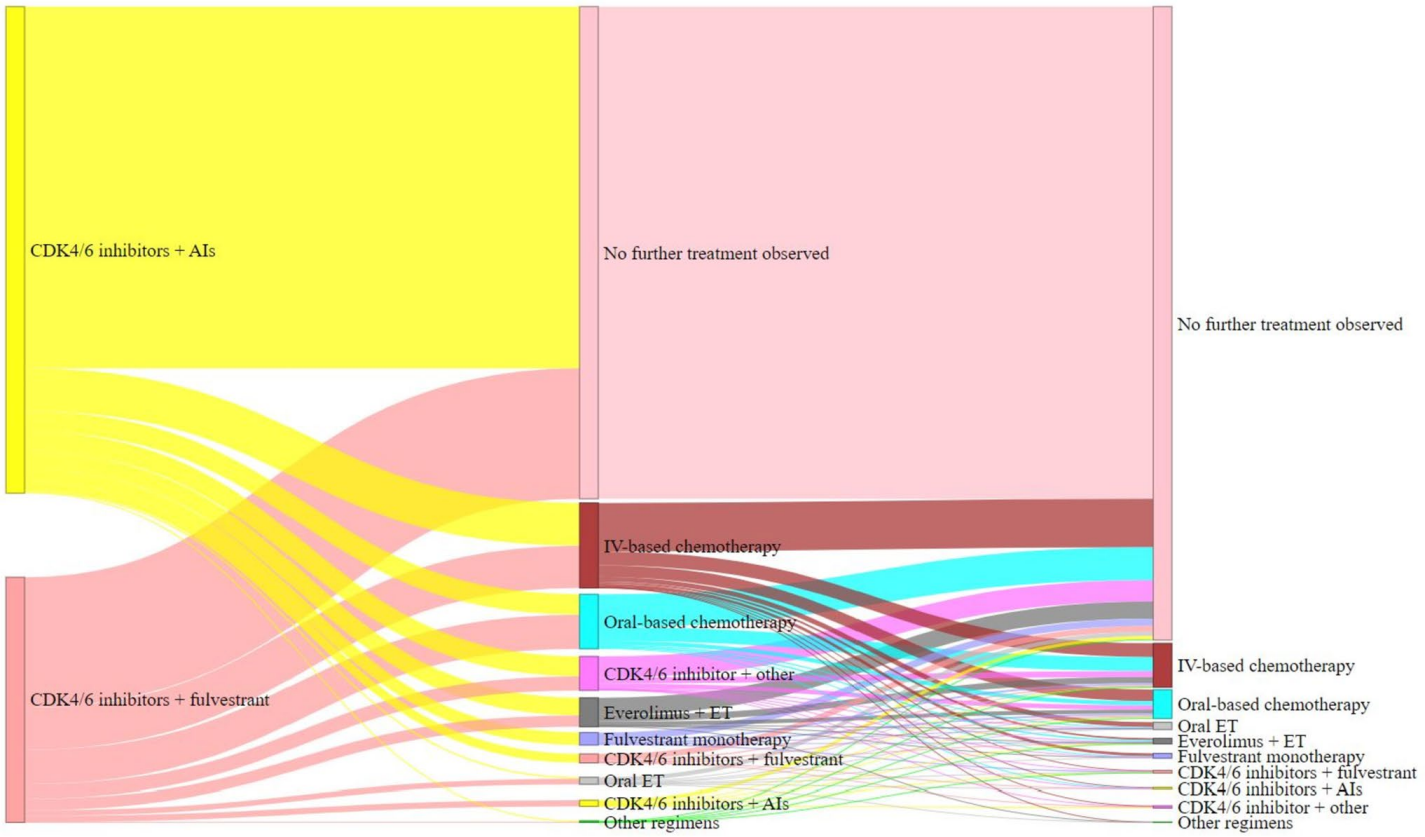
Treatment sequences for patients who received a CDK4/6 inhibitor-based regimen of interest as first line HR+/HER2- mBC treatment. The ‘no further treatment observed’ group comprises patients who did not receive subsequent therapy following initiation of first line treatment before their death or the end of their follow-up

Among patients who received CDK4/6 inhibitors + AIs in first line, 57.8% were still receiving first line treatment at end of follow-up while 25.7% moved to a second line treatment during the follow-up period. The remainder either died or the end of study period was reached before the initiation of subsequent therapy was observed. There was considerable heterogeneity in second line and third line treatments (Supplementary Table S3 and Fig. 1).

After CDK4/6 inhibitors + AIs, 50.6% received a chemotherapy-based regimen and 23.1% received another CDK4/6 inhibitor-based regimen during second line.

Of the patients who received CDK4/6 inhibitors + fulvestrant, 26.7% were still receiving first line treatment at end of follow-up while 46.6% patients moved to second line treatment during follow-up. After CDK4/6 inhibitors + fulvestrant, 67.1% received a chemotherapy-based regimen and 17.8% received another CDK4/6 inhibitor-based regimen during second line.

**Fig. 2:**
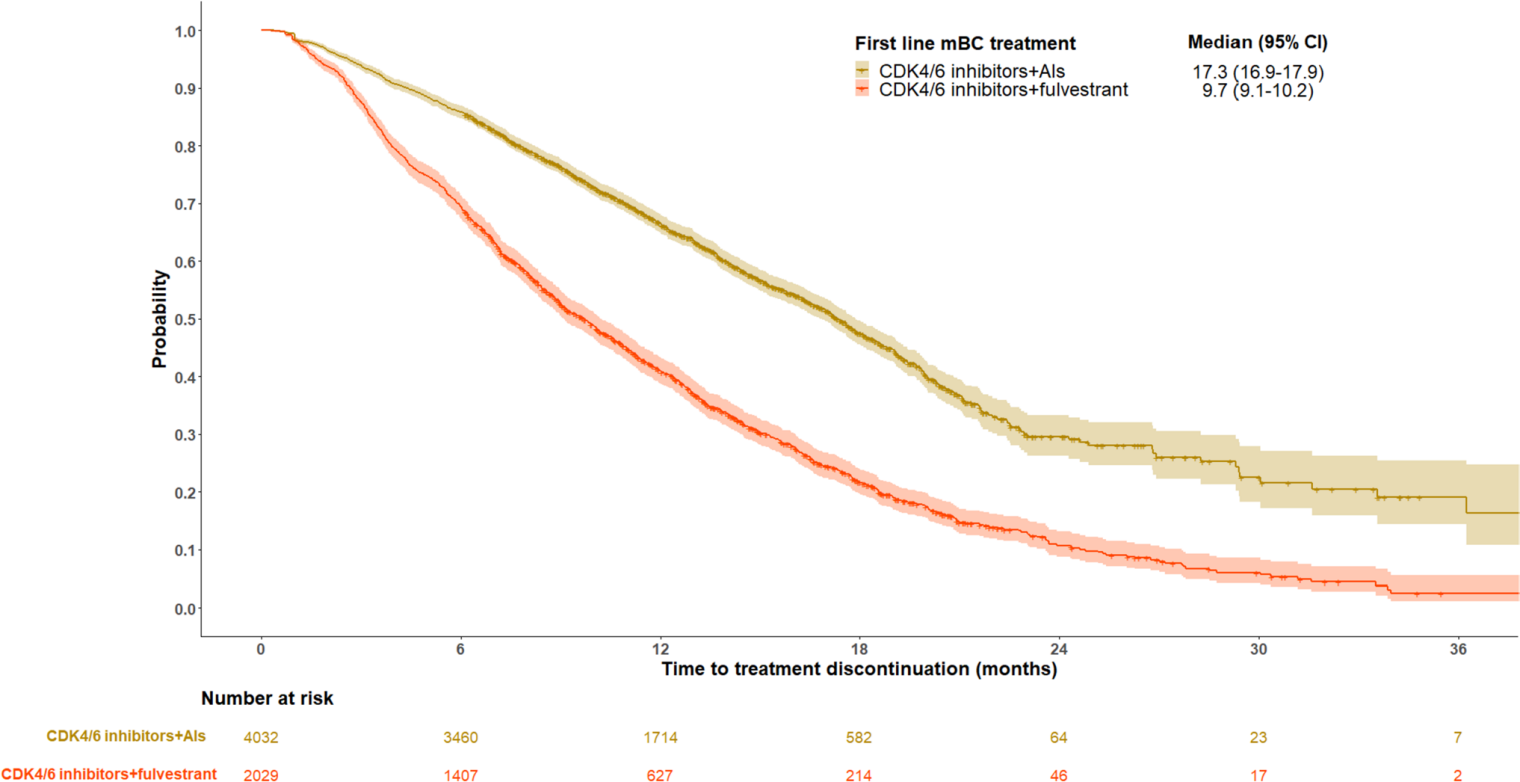
Kaplan–Meier plots of median TTTD for HR+/HER2- mBC patients who received CDK4/6 inhibitors + AIs or CDK4/6 inhibitors + fulvestrant at first line

The median TTTD of first line treatment was 14.2 months (95% CI 13.8–14.6) overall (Fig. 2).

The median TTTD of first line treatment with CDK4/6 inhibitors + AIs was 17.3 months (95% CI 16.9–17.9) and 9.7 months (95% CI 9.1–10.2]) with CDK4/6 inhibitors + fulvestrant. The median TTTD of second line treatment was considerably shorter than first line among patients who received CDK4/6 inhibitors + AIs in first line (4.6 months (95% CI 4.4–4.9)) and among patients who received CDK4/6 inhibitors + fulvestrant in first line (4.7 months (95% CI 4.3–5.1)). TTTD declined further at third line treatment (3.3 months (95% CI 2.8–3.8) for patients treated with CDK4/6 inhibitors + AIs in first line and 3.2 months (95% CI 2.9–3.5) for patients treated with CDK4/6 inhibitors + fulvestrant in first line).

**Fig. 3:**
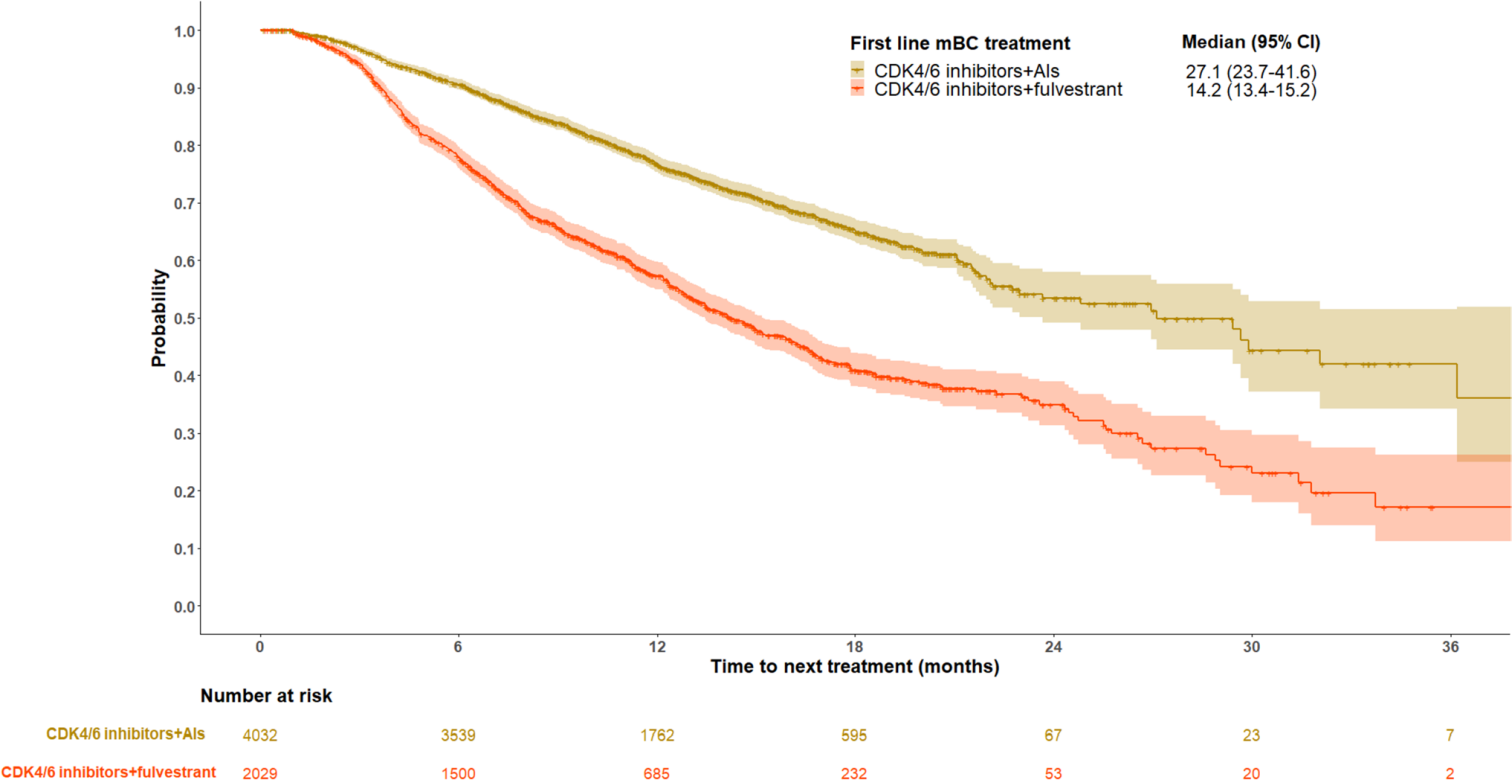
Kaplan–Meier plots of median TTNT for HR+/HER2- mBC patients who received CDK4/6 inhibitors + AIs or CDK4/6 inhibitors + fulvestrant at first line

The median TTNT was 22.2 months (95% CI 21.3–24.5) overall. Median TTNT from first to second line was 27.1 months (95% CI 23.7–41.6) and 14.2 months (95% CI 13.4–15.2) in patients who received CDK4/6 inhibitors + AIs and CDK4/6 inhibitors + fulvestrant at first line, respectively (Fig. 3).

Median TTTD and TTNT were longer in the subgroup of patients defined as endocrine sensitive compared to the full cohort among patients receiving CDK4/6 inhibitors + AIs (TTTD 17.8 months (95% CI 16.8–17.9); TTNT 32.1 months (95% CI 27.1–52.3)) and CDK4/6 inhibitors + fulvestrant (TTTD 11.8 months (95% CI 10.6–12.9); TTNT 21.7 months (95% CI 16.8–27.0)).

## Discussion

### Summary of main findings and relation to other studies

Using a large, national database, this observational real-world study reports patient characteristics, treatment patterns, and outcomes among patients prescribed guideline-recommended first line HR+/HER2- mBC treatment in France. The majority of patients (68%) in the study population received CDK4/6 inhibitors + AIs at first line. Sixty-two percent of CDK4/6 inhibitors + AIs patients and 20.2% of CDK4/6 inhibitors + fulvestrant patients had not received an AI or tamoxifen in the 12 months prior to first evidence of mBC and were assumed to be endocrine sensitive according to the definition used in the present study. For most patients, second line treatment was chemotherapy, though a sizable proportion (14%) were rechallenged with another CDK4/6 inhibitor-based regimen in second line. After a median follow-up of 13.5 months, TTNT was 27.1 months for patients who received CDK4/6 inhibitors + AIs and 14.2 months for patients who received CDK4/6 inhibitors + fulvestrant. Median TTTD was shorter than TTNT, particularly for the CDK4/6 inhibitors + AI group

(CDK4/6 inhibitors + AIs: 17.3 months (95% CI 16.9–17.9); CDK4/6 inhibitors + fulvestrant: 9.7 months (95% CI 9.1–10.2)). These data from the SNDS add to the body of evidence that demonstrates the real-world benefit of CDK4/6 inhibitors and the need for novel treatments once patients experience disease progression.

Guidelines in France did not recommend re-challenge of CDK4/6 inhibitors during the time these data were collected, yet re-challenge was observed in clinical practice. The algorithm used in this study for defining treatment lines does not advance line of therapy if there was a change/switch of the CDK4/6 inhibitors agent or AI. Therefore, re-challenge with CDK4/6 inhibitors was unlikely to be driven by tolerability issues alone. Rather, the findings in this study may reflect deviations from guidelines in clinical practice and re-challenge of a CDK4/6 inhibitor-based regimen as an emerging treatment strategy in France. Limited data are available relating to the effectiveness of re-challenge treatment strategies [19], yet CDK4/6 inhibitor rechallenge patterns have been observed in other real-world data studies. For example, 39.6% of patients who received palbociclib + AI in first line received another CDK4/6 inhibitor-based regimen in second line in a US based real-world study [20]. Moreover, data from the phase 2 MAINTAIN trial demonstrated PFS improvements for patients randomized to fulvestrant or exemestane + ribociclib compared to fulvestrant or exemestane + placebo following progression on a CDK4/6 inhibitor (5.3 months (95% CI 3.3–8.1) vs. 2.8 months (95% CI 2.7–3.3) [21].

PFS among patients with HR+/HER2- mBC who received CDK4/6 inhibitors + AIs has ranged between 20.2 and 27.6 months in clinical trials and 15.1 to 36.7 months in real-world settings [22–27]. For CDK4/6 inhibitors + fulvestrant regimens, the PFS has ranged between 9.5 and 20.5 months in clinical trials and 11.6 and 15.7 months in real-world settings [25, 26, 28]. In the present study, median TTNT falls within these ranges of PFS and TTTD is shorter, likely because patients could discontinue treatment for reasons other than progression (i.e., intolerance/toxicity). Though TTNT and TTTD have been shown to be reasonable proxies of PFS in breast cancer, it is also possible that TTNT overestimates PFS when censoring on death instead of including death as an event [29, 30].

The observed differences in TTNT and TTTD among patients receiving different CDK4/6 inhibitor-based regimens should be interpreted with caution because this is a descriptive study and adjustments were not made for heterogeneity in patient characteristics and treatment history. Patients who received CDK4/6 inhibitors + AI were considerably less likely to have received an AI or tamoxifen in the one year prior to first evidence of mBC (38.2% vs. 79.8% for patients who received CDK4/6 inhibitors + fulvestrant). As such, the CDK4/6 inhibitors + fulvestrant patient group is likely to contain more patients whose disease progressed while receiving or soon after receiving adjuvant ET and may have developed endocrine resistance.

There are two potential explanations for the finding that median TTNT was considerably longer than TTTD, particularly for the CDK4/6 inhibitors + AI group. First, this may be partly explained by the definitions of these outcomes and specifically the way in which deaths were handled. In the TTNT definition, death was a censoring criterion while in the estimation of TTTD, death was considered an event. This difference will have had a direct impact on the calculation of the Kaplan–Meier estimates. Second, there were 4079 patients who did not initiate second line treatment during the follow-up period, including 1206 patients who discontinued first line treatment. These patients who discontinued first line treatment but did not subsequently initiate second line treatment therefore contributed person-time to the estimation of TTNT after the discontinuation of their first line treatment.

As clinical trials and other real-world studies have also shown, TTTD and TTNT in the present study became progressively shorter as patients moved to later lines of therapy [17, 22, 31–34]. In the EMERALD trial the 6-month PFS rates were 34.3% for elacestrant monotherapy and 20.4% for standard of care among patients who had progressed on a CDK4/6 inhibitor-based regimen [34]. These findings along with results from the present study highlight the remaining significant unmet need in HR+/HER2- mBC [20, 35, 36].

### Strengths and limitations

This study used a large, nationwide database to provide contemporary, real-world data on patients with HR+/HER2- mBC.

The study had several limitations. First, some clinical characteristics, laboratory results and inexpensive drugs administered in the hospital were not available from the French SNDS database. Algorithms were developed to identify patients with HR+/HER2- mBC using diagnosis codes on claims as well as treatment-based proxies. It is possible that some patients with early-stage breast cancer or other breast cancer subtypes were misclassified and included in the cohort. The unavailability of data on inexpensive drugs administered in the hospital is not impactful because the drugs of interest in this study are either expensive or dispensed in outpatient settings. Second, the median length of follow-up in this study was short (13.5 months (IQR 9.5–18.1)) due to the relatively recent introduction of CDK4/6 inhibitors, thereby limiting the examination of long-term treatment patterns and outcomes such as mortality. This limitation should be considered when interpreting the findings of this study. Third, date of mBC diagnosis was unavailable within the SNDS and diagnosis codes for secondary malignancies are not reliably used in French clinical practice. As a result, it is possible that the line of therapy algorithm mis-specified the initiation of the first-line regimen and that patients were instead followed from initiation of a later line. For example, suppose a patient received AI monotherapy as first-line mBC treatment and their treating physician never records a diagnosis code for secondary malignancy. If this patient subsequently received a CDK4/6 inhibitor-based regimen in a later line, they would be included in the cohort at this time point and the algorithm would classify the CDK4/6 inhibitor-based regimen as first line. This limitation may explain the relatively high use of AI (29.6% of patients who received CDK4/6 inhibitors + AIs) in the one-year prior to index date. It may also help explain why the patients included in the present study are older than the Epidemio-Strategy Medico-Economical mBC cohort, a retrospective multicenter database which collects data on mBC patients treated in 18 French comprehensive cancer centers (median age: 66.5 vs. 62.0 years, respectively) [5, 16, 37]. The observed proportions of visceral disease in the present study should also be interpreted with caution as they are likely an underestimate of the true prevalence of visceral disease in this patient population due to the under-reporting of codes for secondary malignancies. Nonetheless, other findings were broadly aligned with previous studies, suggesting that the impact of this misclassification may be minimal.

## Conclusions

This study examined real-world patterns of treatment regimens used in the HR+/HER2- mBC setting in France, with a focus on identifying patients treated with guideline recommended first line treatments (CDK4/6 inhibitors + ET).

Overall, these data support the use of CDK4/6 inhibitor-based regimens as an effective first-line therapy for patients with HR+/HER2- mBC. Most patients administered a second line therapy were treated with chemotherapy, thus highlighting an unmet need for novel and effective treatment options in this patient population once their disease has progressed on a CDK4/6 inhibitor-based regimen.

### Supplementary Information

Below is the link to the electronic supplementary material.

Supplementary file1 (DOCX 26 KB)

Supplementary file2 (DOCX 26 KB)

Supplementary file3 (DOCX 25 KB)

Supplementary file4 (DOCX 224 KB)

## Data Availability

Data were shared by the French national health insurance: ‘Caisse Nationale d’Assurance Maladie’ (CNAM).
