# Supplementary material for: Treatment patterns of patients with HR+/HER2- metastatic breast cancer receiving CDK4/6 inhibitor-based regimens: a cohort study in the French nationwide healthcare database
Source: Breast Cancer Res Treat. 2024 Jan 11;204(3):579–88. doi: 10.1007/s10549-023-07201-w (PMC10959771; doi:10.1007/s10549-023-07201-w)
Supplement: Supplementary file 1 — Supplementary file1 (DOCX 26 KB) [file 10549_2023_7201_MOESM1_ESM.docx]

Treatment patterns of patients with HR+/HER2- metastatic breast cancer receiving CDK4/6 inhibitor-based regimens – A Cohort Study in the French nationwide healthcare database

Breast Cancer Research and Treatment

Stephanie H Read^1^, Nadia Quignot^2^, Raissa Kapso-Kapnang^2^, Erin Comerford^3^, Ying Zheng^3^, Corona Gainford^3^, Medha Sasane^3^, Anne-Lise Vataire^4^, Laure Delzongle^4^, Francois-Clement Bidard^5,6^

^1^ Certara UK limited , London, UK

^2^ Certara France, Paris, France

^3^ Sanofi, Cambridge, MA, USA

^4^ Sanofi, Paris, France

^5^ Department of Medical Oncology, Institut Curie, Saint-Cloud, France

^6^ Université Versailles Saint-Quentin, Université Paris-Saclay, Saint-Cloud, France

Corresponding author: Stephanie Read ([Stephanie.Read@certara.com](mailto:Stephanie.Read@certara.com))

Supplementary Table S1. Data sources

| **Data Source** | **Extracted Variables** |
| --- | --- |
| SNIIRAM (claims database) | Patient age |
|  | Patient sex |
|  | Procedures, including type and dates |
|  | Treatments (outpatient), including drug, dose (as per the packaging information), prescription date |
|  | Long-term disease registration (for mBC patient identification and Charlson comorbidity index) |
|  | Vital status |
| PMSI (hospital admissions) | Hospital admissions |
|  | Diagnostic codes (for mBC patient identification, Charlson comorbidity index and visceral disease) |
|  | Procedures (radiotherapy, surgery) |
|  | Expensive drugs dispensed during hospitalization, including drug, amount administered, dispensation date |
| CépiDc (death registry) | Date of death (if applicable) |
|  | Cause of death (if applicable) |
