# Supplementary material for: Treatment patterns of patients with HR+/HER2- metastatic breast cancer receiving CDK4/6 inhibitor-based regimens: a cohort study in the French nationwide healthcare database
Source: Breast Cancer Res Treat. 2024 Jan 11;204(3):579–88. doi: 10.1007/s10549-023-07201-w (PMC10959771; doi:10.1007/s10549-023-07201-w)
Supplement: Supplementary file 2 — Supplementary file2 (DOCX 26 KB) [file 10549_2023_7201_MOESM2_ESM.docx]

Treatment patterns of patients with HR+/HER2- metastatic breast cancer receiving CDK4/6 inhibitor-based regimens – A Cohort Study in the French nationwide healthcare database

Breast Cancer Research and Treatment

Stephanie H Read^1^, Nadia Quignot^2^, Raissa Kapso-Kapnang^2^, Erin Comerford^3^, Ying Zheng^3^, Corona Gainford^3^, Medha Sasane^3^, Anne-Lise Vataire^4^, Laure Delzongle^4^, Francois-Clement Bidard^5,6^

^1^ Certara UK limited , London, UK

^2^ Certara France, Paris, France

^3^ Sanofi, Cambridge, MA, USA

^4^ Sanofi, Paris, France

^5^ Department of Medical Oncology, Institut Curie, Saint-Cloud, France

^6^ Université Versailles Saint-Quentin, Université Paris-Saclay, Saint-Cloud, France

Corresponding author: Stephanie Read ([Stephanie.Read@certara.com](mailto:Stephanie.Read@certara.com))

Supplementary Table S2. Algorithm for defining LOT in patients with HR+/HER2- mBC

| **Variable** | **Operational definition** |
| --- | --- |
| **LOT** | Among incident patients, LOT was defined according to an algorithm using the earliest treatment records following index date. First line of therapy started upon initiation of one of the mBC treatments listed above and included all other treatments indicated for mBC observed within 30 days of the first observed treatment. LOT can begin on the index date.  LOT was continued until the earliest of the following events (LOT end):   - Change in regimens defined as the following: - Treatment Augmentation - A new HR+/HER2- therapy not included in the previous LOT is observed in addition to claims for regimen(s) in the previous LOT within 30 days of the new agent. The augmentation date is the start of the new LOT. - Treatment switch - To qualify for a treatment switch, a claim for a new HR+/HER2- therapy must be observed with no further claims for the original treatment observed after the new treatment has started. The switch date is the start date of the new LOT. - Exceptions - Substitution of an unspecified chemotherapy by an unspecified chemotherapy does not advance the LOT. - Chemotherapy suspended and the same agents later restarted within 90 days does not advance the LOT - Addition or a change of AIs to a line containing AIs does not advance the LOT (as advised by KOL) - Addition or a change of CDK4/6 inhibitors to a line containing CDK4/6 inhibitors does not advance the LOT (as advised by KOL) - If regimens in subsequent LOT are the same as regimens in prior LOT, then combine - Treatment discontinuation (defined below) - Death - Last date of continuous enrollment - End of data availability   The start of new treatment following the end of the LOT using the events described above, if applicable, was considered as a new LOT.  The same approach was used to identify the second line and third line therapy.  Discontinuation was defined as no evidence of a given drug reimbursement during the follow-up period. |

*AI, aromatase inhibitor; CDK4/6, cyclin-dependent kinase 4/6; HR+/HER2-, hormone receptor positive/human epidermal growth factor receptor 2 negative; KOL, key opinion leader; LOT, line of therapy; mBC, metastatic breast cancer*
