# Supplementary material for: Treatment patterns of patients with HR+/HER2- metastatic breast cancer receiving CDK4/6 inhibitor-based regimens: a cohort study in the French nationwide healthcare database
Source: Breast Cancer Res Treat. 2024 Jan 11;204(3):579–88. doi: 10.1007/s10549-023-07201-w (PMC10959771; doi:10.1007/s10549-023-07201-w)
Supplement: Supplementary file 3 — Supplementary file3 (DOCX 25 KB) [file 10549_2023_7201_MOESM3_ESM.docx]

Treatment patterns of patients with HR+/HER2- metastatic breast cancer receiving CDK4/6 inhibitor-based regimens – A Cohort Study in the nationwide Healthcare French database

Breast Cancer Research and Treatment

Stephanie H Read^1^, Nadia Quignot^2^, Raissa Kapso-Kapnang^2^, Erin Comerford^3^, Ying Zheng^3^, Corona Gainford^3^, Medha Sasane^3^, Anne-Lise Vataire^4^, Laure Delzongle^4^, Francois-Clement Bidard^5,6^

^1^ Certara UK limited , London, UK

^2^ Certara France, Paris, France

^3^ Sanofi, Cambridge, MA, USA

^4^ Sanofi, Paris, France

^5^ Department of Medical Oncology, Institut Curie, Saint-Cloud, France

^6^ Université Versailles Saint-Quentin, Université Paris-Saclay, Saint-Cloud, France

Corresponding author: Stephanie Read (Stephanie.Read@certara.com)

Supplementary Table S3. Ten most frequent treatment regimens during complete follow up by LOT – Main cohort of 6,061 patients who received a CDK4/6 inhibitors-based regimen of interest as first line HR+/HER2- mBC treatment

| **Most frequent treatment regimens** | **Overall** | **Patients who received CDK4/6 inhibitors + AIs** | **Patients who received CDK4/6 inhibitors + fulvestrant** |
| --- | --- | --- | --- |
|  | **No. of patients (% among the number of patients within the LOT)** | | |
| **LOT 1 (N=6,061)** | | | |
| CDK4/6 inhibitors + AIs | 4032 (66.5) | 4032 (100.0) | NA |
| CDK4/6 inhibitors + fulvestrant | 2029 (33.5) | NA | 2029 (100.0) |
| **LOT 2 (N=1,982)** | | | |
| IV chemotherapy | 597 (30.1) | 288 (27.8) | 309 (32.7) |
| Oral chemotherapy | 403 (20.3) | 147 (14.2) | 256 (27.1) |
| CDK4/6 inhibitors + other therapy | 282 (14.2) | 165 (15.9) | 117 (12.4) |
| Everolimus and endocrine therapy | 239 (12.1) | 145 (14.0) | 94 (9.9) |
| Fulvestrant monotherapy | 105 (5.3) | 105 (10.1) | <10 |
| CDK4/6 inhibitors + fulvestrant | 74 (3.7) | 74 (7.1) | <10 |
| AI and IV chemotherapy | 54 (2.7) | 53 (5.1) | <10 |
| CDK4/6 inhibitors + AIs | 51 (2.6) | 0 | 51 (5.4) |
| AI monotherapy | 36 (1.8) | 0 | 36 (3.8) |
| Fulvestrant + IV chemotherapy | 25 (1.3) | <10 | >20 |
| AI + oral chemotherapy | <10 | 18 (1.74) | <10 |
| Tamoxifen monotherapy | <10 | 11 (1.06) | 10 (1.1) |
| IV chemotherapy + oral chemotherapy | <10 | <10 | 12 (1.3) |
| Oral chemotherapy + fulvestrant | <10 | <10 | 22 (2.3) |
| **LOT 3 (N=809)** | | | |
| IV chemotherapy | 322 (39.9) | 140 (36.9) | 182 (42.5) |
| Oral chemotherapy | 214 (26.5) | 94 (24.7) | 120 (28.0) |
| Everolimus + endocrine therapy | 46 (5.7) | 20 (5.3) | 26 (6.1) |
| AI monotherapy | 43 (5.3) | 18 (4.7) | 25 (5.8) |
| Fulvestrant monotherapy | 41 (5.1) | 26 (6.8) | 15 (3.5) |
| CDK4/6 inhibitors + fulvestrant | 21 (2.6) | 14 (3.78) | <10 |
| CDK4/6 inhibitors + other therapy | 18 (2.2) | <10 | 12 (2.80) |
| Tamoxifen monotherapy | 16 (2.0) | 11 (2.9) | <10 |
| IV chemotherapy + oral chemotherapy | 14 (1.7) | <10 | <10 |
| CDK4/6 inhibitors + AI | 14 (1.7) | <10 | <10 |
| AI, chemotherapy + fulvestrant or tamoxifen | <10) | <10 | <10 |
| AI + oral chemotherapy | <10 | <10 | <10 |

*AI, aromatase inhibitor; CDK4/6, cyclin-dependent kinase 4/6; HR+/HER2-, hormone receptor positive/human epidermal growth factor receptor 2 negative; IV,* *Intravenous; LOT, line of therapy; mBC, metastatic breast cancer; NA, not applicable*
