# Supplementary material for: Treatment patterns of patients with HR+/HER2- metastatic breast cancer receiving CDK4/6 inhibitor-based regimens: a cohort study in the French nationwide healthcare database
Source: Breast Cancer Res Treat. 2024 Jan 11;204(3):579–88. doi: 10.1007/s10549-023-07201-w (PMC10959771; doi:10.1007/s10549-023-07201-w)
Supplement: Supplementary file 4 — Supplementary file4 (DOCX 224 KB) [file 10549_2023_7201_MOESM4_ESM.docx]

Treatment patterns of patients with HR+/HER2- metastatic breast cancer receiving CDK4/6 inhibitor-based regimens – A Cohort Study in the French nationwide healthcare database

Breast Cancer Research and Treatment

Stephanie H Read^1^, Nadia Quignot^2^, Raissa Kapso-Kapnang^2^, Erin Comerford^3^, Ying Zheng^3^, Corona Gainford^3^, Medha Sasane^3^, Anne-Lise Vataire^4^, Laure Delzongle^4^, Francois-Clement Bidard^5,6^

^1^ Certara UK limited, London, UK

^2^ Certara France, Paris, France

^3^ Sanofi, Cambridge, MA, USA

^4^ Sanofi, Paris, France

^5^ Department of Medical Oncology, Institut Curie, Saint-Cloud, France

^6^ Université Versailles Saint-Quentin, Université Paris-Saclay, Saint-Cloud, France

Corresponding author: Stephanie Read (Stephanie.Read@certara.com)


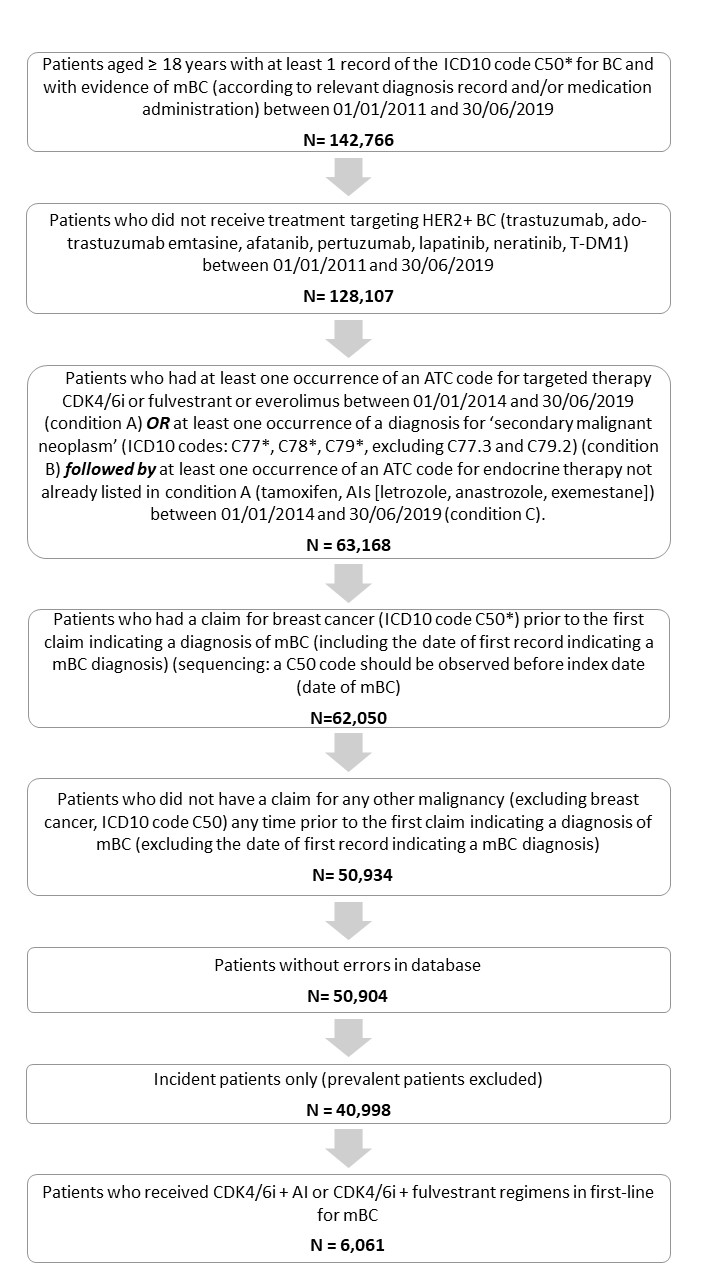


Supplementary Fig. S1 Study flow chart for overall cohort

*AI, aromatase inhibitor; ATC, Anatomical Therapeutic Chemical; BC, breast cancer; CDK4/6i, cyclin-dependent kinase 4/6 inhibitor; HER2+, human epidermal growth factor receptor 2 positive; ICD10, International Classification of Diseases 10^th^ revision; mBC, metastatic breast cancer*
